# Facile Synthesis of Platinum Nanoparticle-Embedded Reduced Graphene Oxide for the Detection of Carbendazim

**DOI:** 10.3390/ma16247622

**Published:** 2023-12-13

**Authors:** Suthira Pushparajah, Shinichi Hasegawa, Tien Song Hiep Pham, Mahnaz Shafiei, Aimin Yu

**Affiliations:** School of Science, Computing, and Engineering Technology, Swinburne University of Technology, Hawthorn, VIC 3122, Australia; spushparajah@swin.edu.au (S.P.); songhiepbt@yahoo.com (T.S.H.P.); mshafiei@swin.edu.au (M.S.)

**Keywords:** carbendazim, electrochemical sensors, electrodeposition, platinum nanoparticles, reduced graphene oxide

## Abstract

In recent years, there has been a significant interest in the advancement of electrochemical sensing platforms to detect pesticides with high sensitivity and selectivity. Current research presents a novel approach utilising platinum nanoparticles (NPs) and reduced graphene oxide deposited on a glassy carbon electrode (Pt-rGO/GCE) for direct electrochemical measurement of carbendazim (CBZ). A straightforward one-step electrodeposition process was applied to prepare the Pt-rGO sensing platform. The incorporation of conductive rGO nanosheets along with distinctive structured Pt NPs significantly enhanced the effective electrode surface area and electron transfer of CBZ. Additionally, when exposed to 50 µM CBZ, Pt-rGO/GCE exhibited a higher current response compared to the bare electrode. Further investigations were performed to analyse and optimise the experimental parameters that could influence pesticide detection. Under the optimised conditions of pH 7 and 5 min of accumulation time, the Pt-rGO/GCE sensor showed a linear concentration detection range from 0.1 µM to 50 µM, with a detection limit of 3.46 nM. The fabricated sensor was successfully employed for CBZ detection in milk and tap water with 98.88% and 98.57% recovery, respectively. The fabricated sensor showed higher sensitivity and reproducibility, thus indicating the potential of this technology in the development of reliable sensors for the detection of CBZ or similar pesticides in forthcoming applications.

## 1. Introduction

The most widely used synthetic, systemic benzimidazole fungicide, known as carbendazim (CBZ) (C_9_H_9_N_3_O_2_), has long-lasting efficacy for preventing and eliminating pathogenic fungi (e.g., *Ascomycetes*, *Fungi imperfecti* and *Basidiomycetesin*) in crops including cereals, vegetables, and fruits [1,2]. However, exposure to CBZ may develop germ cell apoptosis, embryotoxicity teratogenesis, infertility, developmental toxicity, hepatocellular dysfunction, disruption of haematological processes, and mutagenicity of important mammalian species [3,4]. As part of the regulation (EC No 396/2005) for the guarantee of food quality, the European Union (EU) has specified maximum residual limits (MRLs) of pesticides that may be found in food items. MRLs for CBZ, for instance, in citrus varied from 100 to 700 ppb (Risk Assessment for Safety of Orange Juice Containing Fungicide Carbendazim, 2012) [5]. Due to the potential negative impacts previously mentioned and proper quantity control of CBZ in the food industry, developing sensitive pesticide detection systems is of the utmost importance and requires immediate attention to ensure the protection of human health and the environment.

Over the years, common detection techniques included high-performance liquid chromatography (HPLC) [6], gas chromatography [7], and liquid chromatography coupled with mass spectrometry (LC-MS) [8], which have been applied to detect CBZ pesticide residues. Although HPLC and gas chromatography–mass spectrometry have good sensitivity and selectivity, their sample preparation procedures are often complicated and time-consuming [9]. As alternatives, voltammetric and fluorescence measurements are less costly than capillary electrophoresis with mass spectrometry or HPLC [10]. For this reason, electrochemical methods have recently gained attention for electroactive pesticide detection as a result of their high sensitivity, simplicity, rapid detection and portability [11]. Additionally, their sensitivity could be further improved by modifying the sensor with functional materials, including carbon materials [12], natural enzymes [13], polymers [14], noble metal nanoparticles [15], and metal oxides [16]. In recent research, considerable attention has been given to metal nanoparticles (NPs) due to their distinctive structures and inherent characteristics.

Among various metal NPs, platinum NPs display high electroconductivity and electrocatalytic activity for the signal amplification of electrochemical sensors [17]. However, Pt NPs are prone to significant agglomeration, which negatively impacts their catalytic performance. To address this issue and achieve better dispersibility, Pt NPs are often deposited onto carbon support materials [18]. For electrocatalytic purposes, these carbon support materials should possess a high surface area, abundant binding sites, excellent stability, and strong electrical conductivity. Among various options, two-dimensional (2D) carbon-based materials like graphene and its derivative (e.g., reduced graphene oxide (rGO)) are commonly employed due to their ability to fulfill the aforementioned requirements [19].

In this work, a combined nanocomposite of Pt NPs and rGO has been successfully fabricated as a promising electrode modifier to enhance the electrochemical detection of CBZ. Both Pt and rGO are electro-deposited onto the surface of a glassy carbon electrode (GCE) utilising the cyclic voltammetry (CV) method. The experimental results have shown that existing oxygen-containing functional groups in rGO could facilitate the uniform nucleation and growth process of Pt NPs and effectively prevent aggregation. On the other side, the electrodeposition of Pt NPs on the surface of rGO would significantly increase the active electrode surface area. Following the material characterization through various methods, we have extensively investigated the detecting capabilities of the Pt-rGO composite-modified GCE towards CBZ using electrochemical techniques. Hence, this research aims to develop an innovative sensing platform with improved sensitivity and a faster response time. This will be achieved through the application of a one-step electrodeposition technique, marking the novelty of this study.

## 2. Materials and Methods 

### 2.1. Materials

All experiments utilised chemicals of analytical grade, which were employed without undergoing any additional purification. Carbendazim (CBZ, 97%), potassium hexacyanoferrate (II) trihydrate (K_4_[Fe(CN)_6_.3H_2_O), platinum (II) chloride (metal basis 98%, PtCl_2_), sodium chloride (NaCl), ammonium sulfate (NH_4_)_2_SO_4_, potassium iodide (KI), monobasic sodium phosphate (NaH_2_PO_4_), dibasic sodium phosphate (Na_2_HPO_4_) and potassium nitrate (KNO_3_) were purchased from Sigma-Aldrich (Darmstadt, Germany). Graphene oxide (GO) powder was acquired from JCNANO, Inc. (Nanjing, China). D-glucose (AR-grade), hydrochloric acid (HCl) and sodium hydroxide (NaOH) pellets were obtained from Merck Pty Ltd. (Darmstadt, Germany). Glassy carbon electrode (GCE, (3 mm in diameter), Ag/AgCl (3M KCl) electrode, a platinum wire electrode and gamma alumina powder (1.0, 0.3, 0.05 µm) were obtained from Gaoss Union Company (Wuhan, China). A mixture of 0.1 M Na_2_HPO4 and NaH_2_PO_4_ was used to prepare phosphate buffer solutions (PBS). The solution’s pH was adjusted with 0.1 M HCl or 0.1 M NaOH solutions. Milli-Q water (18.2 MΩ cm) was utilised throughout all experiments.

### 2.2. Preparation of Pt NPs and rGO Modified GCE via One-Step Electrodeposition

Prior to electrodeposition, the GCE was subjected to a 3 min wash in an ultrasonic bath filled with water. Subsequently, a slurry containing alumina particles (1 µm) was employed to perform the polishing of GCE, which was then subjected to the 3 min ultrasonic cleaning procedure with water. The procedure was repeated utilising alumina slurries of sizes 0.3 µm and 0.05 µm, respectively. After polishing, the electrodes were sequentially cleaned using 100% ethanol followed by water through two rounds of 3 min ultrasonic baths. The freshly cleaned GCE was immersed into the GO dispersion (1.0 mg/mL) or PtCl_2_ solution (0.5 mg/mL) to prepare rGO/GCE and Pt/GCE, respectively. The electrodeposition procedure was conducted using the CV method at a scan rate of 0.1 V/s. The potential scan was repeatedly cycled from−1.5 to 1.0 V (rGO/GCE) and −1.1 to 1.0 V (Pt/GCE) for 10 cycles. Once the electrodeposition was completed, the rGO/GCE and Pt/GCE were washed with water and left to air dry. Similarly, Pt-rGO/GCE was obtained through the electrodeposition of the cleaned GCEs in a mixed GO and PtCl_2_ solution. For comparison purposes, the bare GCE was prepared using a similar step, however without any material electrodeposited onto its surface. All modified electrodes and the bare electrode were kept at room temperature for future electrochemical tests.

### 2.3. CBZ Sample Preparation

CBZ powder (97%, supplied from Sigma-Aldrich, (Darmstadt, Germany)) was dissolved in water to prepare a 500 µM CBZ stock solution. To prepare CBZ samples in skim milk and tap water, tap water and skim milk (purchased from Coles supermarket, Melbourne, Australia) underwent initial filtration to eliminate any suspended solids. A mixture of PBS (pH of 7.0): tap water/skim milk (1:5) was prepared as the electrochemical testing medium before CBZ stock solutions were added. The presence of CBZ in skim milk and tap water was detected using the differential pulse voltammetry (DPV) assay.

### 2.4. Electrochemical Measurements and Characterization of the As-Prepared GCEs

CHI660 Electrochemical workstation (Champaign, IL, USA) and a conventional three-electrode setup were used to perform the electrochemical measurements. The three-electrode setup consisted of a platinum wire as an auxiliary electrode, an Ag/AgCl reference electrode (3M KCl), and a working electrode (either a bare or modified GCE). The voltage used for the CV tests ranged from 0.2 to 1.2 V to confirm the existence of CBZ. DPV measurements were executed with an amplitude of 0.05 V and a pulse period of 0.5 s within the range from 0.6 to 1.1 V. CV scans and electrochemical impedance spectroscopy (EIS) studies were carried out on all modified GCEs and bare GCE. These tests were performed in a 0.1 M KCl solution with 1.0 mM K_4_[Fe(CN)_6_]. The EIS measurements were recorded between the frequencies of 10^−2^ and 10^5^ Hz when the bias potential was set to 0.2 V. The structural morphology and elemental compositions of each GCE were examined using a ZEISS SUPRA 40 PV scanning electron microscope (SEM) combined with energy-dispersive X-ray spectroscopy (EDX) (Oberkochen, Germany). The FTIR spectra were acquired with the Nicolet iS5 type FTIR instrument (Waltham, MA, USA) to investigate the functional groups in GO and rGO.

## 3. Results and Discussion

### 3.1. Electrodeposition of rGO onto GCE

**Figure 1:**
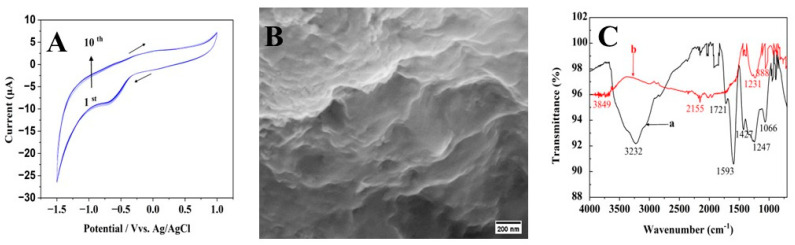
(**A**) Electrodeposition of GO at the bare GCE for 10 CV cycles at 0.1 V/s (arrow indicates potential scanning direction). (**B**) SEM image of electrodeposited rGO/GCE and (**C**) FTIR spectra of (a) GO and (b) electrodeposited rGO.

Figure 1A illustrates the typical cyclic voltammograms (CVs) of rGO electrochemical deposition on GCE at 0.1 V/s. Two peaks, including one reduction and one oxidation, can be observed in the CV profile. The slight oxidation peak obtained at −0.11 V may be explained by the fact that some oxygen functional groups on the graphene surfaces of GO sheets were oxidized [20]. These groups are electrochemically active and remain stable, rendering them resistant to reduction using the CV technique. The reduction peak detected at −0.61 V during the reverse scan may be responsible for the reduction in GO sheets [21]. Electrochemically reducing GO formed rGO sheets, which quickly attached to the electrode’s surface. This is proved with further potential scans; reduction peak and oxidation peak currents have continued to rise, proving that conducting graphene has been successfully deposited on the GCE [21].

The surface morphology of the electrodeposited rGO/GCE (Figure 1B) was examined using SEM. The rGO/GCE displayed regions with folding and wrinkles [22]. Additionally, EDX analysis (Figure S1A) validates the presence of carbon (C) and oxygen (O) on the electrode surface. Figure 1C depicts the FTIR spectra of the GO’s chemical composition both prior to and following reduction. To reduce GO, functional groups that contain oxygen must be removed, and conjugated π systems must be restored. The GO spectrum (curve a) shows distinct peaks associated with the following functional groups, including C-O (1066 cm^−1^, C-O-C (1247 cm^−1^), C-OH (1427 cm^−1^), C=C (1593 cm^−1^), C=O (1721 cm^−1^) and -OH (3232 cm^−1^). The disappearance of these bands on rGO (curve b) indicates that GO has undergone a successful chemical reduction. For example, the absence of the formation of hydrogen bonds by carboxyl (COOH) (3232 cm^−1^) groups implies a noteworthy reduction in the OH band within rGO [23,24]. A significant number of crystallographic flaws present in GO’s basal plane cause the appearance of oxygenated functional groups on its surface [25]. 

### 3.2. One-Step Electrodeposition of Pt NPs and rGO onto GCE

**Figure 2:**
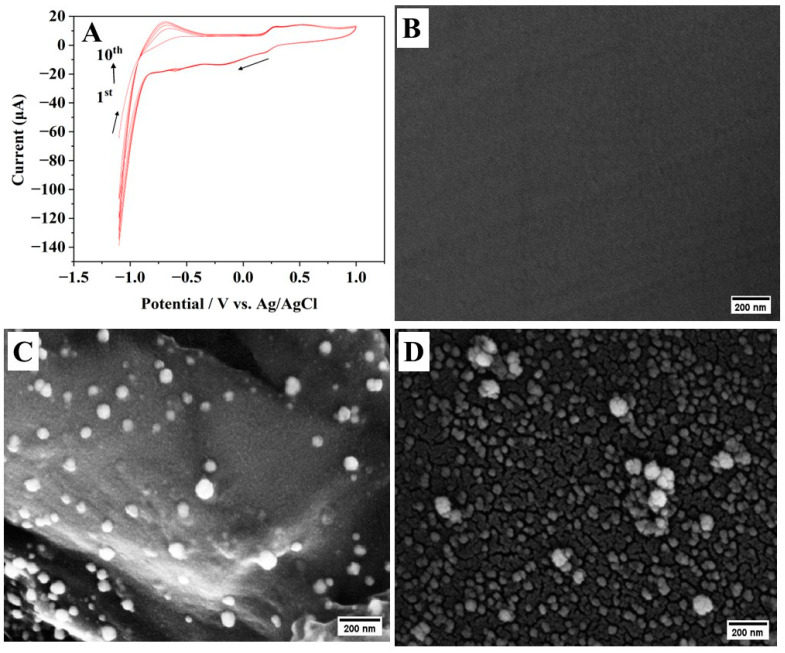
(**A**) Electrodeposition of GO and Pt onto bare GCE using CV at a scan rate of 0.1 V/s (arrow indicates potential scanning direction). SEM images of (**B**) bare GCE, (**C**) electrodeposited Pt-rGO/GCE and (**D**) electrodeposited Pt-GCE.

By adding platinum salt to the GO dispersion, rGO and Pt NPs are simultaneously electrodeposited onto GCE using the same CV approach to achieve the formation of Pt-rGO/GCE. Figure 2A illustrates Pt and rGO deposition during the repeated CV scan for 10 cycles. The initial scan was started at −1.1 V to induce the formation of Pt nuclei by reducing the Pt^2+^. One distinct reduction peak is detected at −0.275 V during the reverse scan. The observed reduction peak is due to Pt^2+^ being electrochemically reduced to Pt, indicating that the potential exceeds the reduction potential of Pt^2+^. Simultaneously, the reduction in hydrogen ions led to the evolution of hydrogen gas. The presence of the oxidation peak at +0.26V is closely related to the oxidation of produced hydrogen gas. Finally, it is apparent that there is a significant rise in maximum current seen during the electrodeposition of Pt and rGO with each successive cycle. This observation implies that the Pt NPs and rGO are effectively deposited on the surface of the GCE. 

For further investigations, SEM was used to examine the surface morphology of the as-prepared Pt-rGO/GCE. As shown in Figure 2B, bare GCE has a smooth surface. Following the electrodeposition of rGO and Pt NPs, a significant alteration occurred (Figure 2C). Further, the EDX spectrum (Figure S1B) verifies that these particles indeed consist of C, O and Pt. In particular, the Pt-rGO/GCE shows a more textured surface because of the agglomeration of rGO sheets, which act as a conductive matrix, providing a consistent foundation for numerous smooth and round-shaped Pt particles to securely attach themselves. The average size of Pt particles in Pt-rGO/GCE is found to be 58 nm (Figure S1D).

For comparison, the electrodeposition of Pt NPs without GO was also studied, and the SEM image of the surface morphology of the Pt/GCE is shown in Figure 2D. The EDX spectrum (Figure S1C) verifies that these particles indeed consist of C and Pt. In the absence of rGO, the average Pt particle size is 87 nm (Figure S1E). However, some aggregation of Pt particles was seen to form a cauliflower shape. Therefore, the presence of GO could effectively prevent particle aggregation during its growth. Also, the addition of GO has resulted in a more uniform distribution of Pt particles on the electrode surface compared to Pt/GCE. This uniformity is desirable for achieving consistent and reliable electrochemical performance. Further, this reduction in particle size can be advantageous for various reasons, including increased surface area and improved electrocatalytic activity.

### 3.3. Electrochemical Behaviour of Modified Electrodes

**Figure 3:**
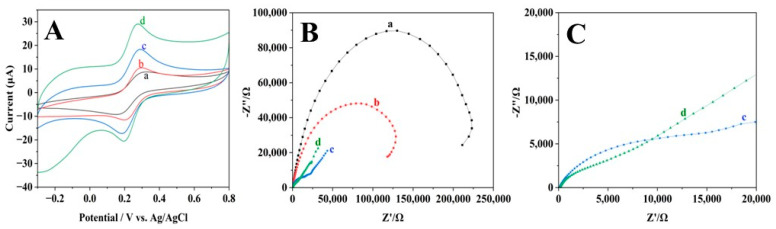
(**A**) CV graphs and (**B**) Nyquist plots of EIS of the (a) bare GCE, (b) rGO/GCE, (c) Pt/GCE and (d) Pt-rGO/GCE. (**C**) A closer examination of the Nyquist plots from the EIS of (c) Pt/GCE and (d) Pt-rGO/GCE in 0.1 M KCl solution containing 1.0 mM K_4_[Fe(CN)_6_].

The electrochemical behaviour of the modified electrodes was first studied using the CV method with K_4_[Fe(CN)_6_] as a redox probe (shown in Figure 3A). The quantity of electrons transferred from the electrode to the redox indicator is directly proportional to the peak current observed in the CV curve. Consequently, the peak current will increase as the electrode’s efficiency increases. It was evident from the results that [Fe(CN)_6_]^4−^ underwent a reversible redox phenomenon at all electrodes, as each electrode exhibited two distinct redox peaks between −0.3 and 0.8 V. Further, it is obvious that modified electrodes provide improved current responses than the bare GCE. In comparison to bare GCE, the Pt-rGO/GCE shows a superior performance, as evidenced by a significantly higher peak current intensity, which has increased by a factor of 2.5. In addition, the effective surface area for the various GCE modifications can be calculated by using the Randles–Sevcik formula [26], which can be found below. Our underlying assumption was that the diffusion process was the only mechanism for mass transportation.

(1) $$Ip=2.69\times 10^{5}\;AD^{1/2}\;n^{3/2}\;V^{1/2}\;C$$

*A*: The electroactive surface area of the electrode (cm^2^)
*D*: Diffusion coefficient of the molecule (cm^2^/s) (K_4_[Fe(CN)_6_] = 7.6 × 10^-6^ cm^2^/s)
*n*: Number of electron responsible for redox reaction (*n* = 1)
*V*: Scan rate (V/s)
*C*: Concentration of the redox probe (mol/cm^3^)

The electroactive surface areas for the various GCE modifications are determined using Equation (1). The calculated values of electroactive surface areas are as follows: bare GCE (0.0324 cm^2^), rGO/GCE (0.0424 cm^2^), Pt/GCE (0.0678 cm^2^), Pt-rGO/GCE (0.0721 cm^2^). Increased redox peak current is correlated with enhanced electrode surface area [27]. Furthermore, the Pt-rGO/GCE composite significantly changed the surface of the bare electrode, displaying a 2.2-fold increase in surface area over that of the bare GCE. The values of the peak potential differences (ΔE*p*) for the following electrodes are estimated as follows: bare GCE (0.324 V), rGO/GCE (0.297 V), Pt/GCE (0.290 V), and Pt-rGO/GCE (0.232 V). The reduction in ΔE*p* signifies that the Pt and rGO electrodeposited onto the electrode surface effectively promotes the transfer of electrons, leading to amplified current sensitivity and a raised detection threshold for the modified electrode [28].

EIS was further used to investigate the electron transport characteristics of various modified electrodes. The electron transfer process in EIS measurements is hindered by the semicircle portion of the Nyquist plot at higher frequencies. The electron transfer resistance (also known as Ret) corresponds to the diameter of the semicircle that runs along Z’, whereas the linear section observed at lower frequencies denotes the existence of Warburg diffusion resistance [29]. In this study, EIS was employed to examine the fluctuations in the impedance of the electrode surfaces. Every semicircular step illustrated in the graph corresponds to the electrochemical process of electro-electric charge transfer involving K_4_[Fe(CN)_6_], which occurs on the surface of the modified electrodes. The EIS measurements of four different electrodes are illustrated in Figure 3B. The electrochemical properties of the working electrodes, which have been modified with rGO and Pt particles, were investigated and analysed.

Obviously, a larger interface electron transfer resistance Ret value of ~235,000 Ω was obtained at the bare GCE (Figure 3B, curve a). It is possible that this is because the bare GCE surface has an obstruction effect, which reduces the capacity of the material to transport charge. In contrast to the bare electrode, there was a decrease in the Ret values of the modified electrode. Following the deposition of rGO (Figure 3B, curve b), the Ret value of the electrode experienced a decrease of approximately ~148,000 Ω. This observation indicates that the rGO is electrically conductive and has a high surface area, facilitating the electron transport from the electrode to the target. It is obviously seen that Ret value of Pt/GCE continued to drop to ~15,000 Ω, implying that the modified electrode facilitated improved charge transfer and reduced electrical resistance due to the presence of Pt particles with exceptional chemical properties (Figure 3C, curve c). The Nyquist plot for Pt-rGO/GCE is illustrated with (Figure 3C, curve d), exhibiting a smaller semicircle when compared to that of Pt/GCE. This suggests that Ret has decreased while conductivity has increased. In contrast to Pt/GCE, the Pt-rGO combination demonstrated the enhanced sensing capabilities of modified GCE in terms of K_4_[Fe(CN)_6_]. This observation can be credited to the Pt NPs with rGO sheets in combination, which synergistically facilitates electron transport between the [Fe(CN)_6_]^4−^ and the modified electrode surface. Most of these findings are consistent with prior CV results.

### 3.4. Electrochemical Behaviour of CBZ at Bare and Modified Electrodes

**Figure 4:**
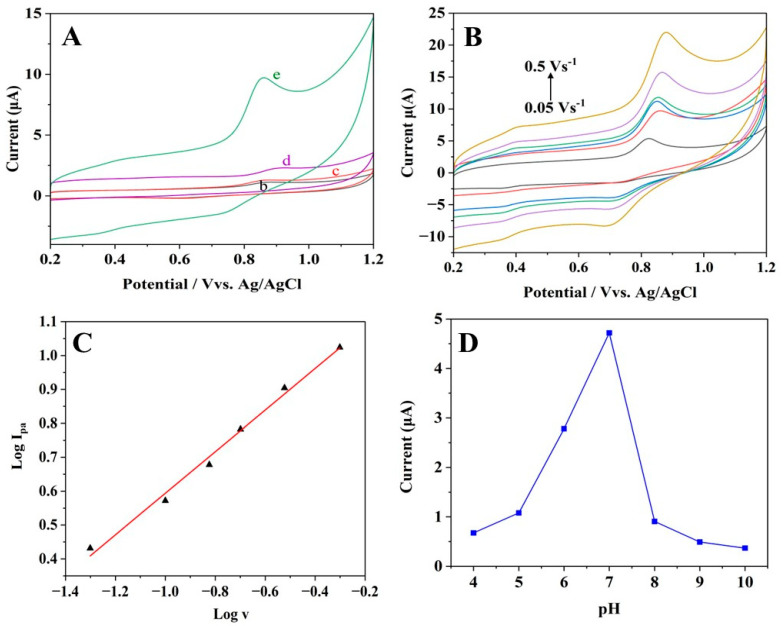
(**A**) CV graphs of (b) bare GCE, (c) rGO/GCE, (d) Pt/GCE and (e) Pt-rGO/GCE in the presence of 50 µM CBZ in pH 7.0 PBS at 0.1 V/s. (**B**) CV scans of 50 µM CBZ in pH 7.0 PBS at different scan rates: 0.05, 0.10, 0.15, 0.20, 0.30 and 0.50 V/s. (**C**) Relationship between the log (Ipa) vs. log (v). (**D**) Current response of 50 µM CBZ at different pH levels.

The CBZ detection ability of the as-prepared bare and modified electrodes was analysed using voltammetric techniques. In the selected potential range from 0.2 to 1.2 V, redox peaks were not identified at the bare GCE when CBZ was not present (Figure S2A, curve a). However, the addition of 50 µM CBZ produced a very weak oxidation peak at 0.87 V (Figure 4A, curve b) on the bare GCE, which resulted from the oxidation of CBZ. Pt and rGO sheets were separately deposited, as shown in Figure 4A. Curve (c) is for rGO/GCE, and (d) is for Pt/GCE, and it was evident that both the background and redox peak current exhibited a notable increase due to the higher active surface area and electrical conductivity. In contrast, Pt-rGO/GCE (Figure 4A, curve e) exhibited a clearly defined increased oxidation peak current along with the presence of 50 µM CBZ, indicating that the inclusion of Pt and rGO sheets improves the electrochemical oxidation of CBZ. Remarkably, the maximum current observed for CBZ at Pt-rGO/GCE (Figure 4A, curve e) was 16 times greater compared to bare GCE (Figure 4A, curve b). There are two potential causes that might be responsible for this outcome. Firstly, both Pt and rGO, together with great electroactive area, effectively increased the amount of CBZ that can be loaded onto the modified electrode surface. Due to the high conductivity of the Pt-rGO composite, the modified electrode and CBZ in solution may transfer electrons more easily, which increases the detection response [30]. Furthermore, it is obvious that the peak current potential during the electrochemical oxidation of CBZ has experienced a negative shift from 0.87 V (bare GCE) to 0.81 V (Pt-rGO/GCE).

The assessment of the kinetics information regarding the chemical process at the electrode surface can be achieved by examining the scan rate effect on the peak current and peak potential of the oxidation. The electrochemical oxidation of CBZ was studied by using the CV method. Figure 4B illustrates the correlation between scan rate and current response observed for 50 µM CBZ at Pt-rGO/GCE. When the scan rate increased from 0.05 to 0.50 V/s, the peak potentials for oxidation (Epa) and peak potentials for reduction (Epc) moved to more positive and negative directions, respectively. Meanwhile, the peak currents during the oxidation and reduction phases (Ipa and Ipc) showed a gradual increase. This may be explained by changes in the kinetic effects of Pt-rGO/GCE towards CBZ electrochemical oxidation. Figure 4C shows the relationship between log (Ipa) and log (v) with accompanying regression equation (R = 0.992), as below:

(2) logIpa=1.2071+0.6134 log v

The slope evident in Equation (2) was 0.6134, suggesting that the electrochemical oxidation of CBZ at the Pt-rGO modified GCE was regulated by both diffusion and adsorption processes. 

We further examined the effect of the pH from 4.0 to 10.0 on the current response for 50 µM CBZ at Pt-rGO/GCE (Figure 4D). According to the results, the oxidation peak current gradually increased from a pH value of 4.0 to 7.0, indicating the electrochemical process of CBZ involves protons [31]. The electrochemical oxidation mechanism of CBZ is depicted in Figure S3 [32]. The fluctuation in pH affects the concentration of protons, which, in turn, influences current response. The pH of the surrounding microenvironment may have a role in the inclusive capturing capacity of Pt and rGO molecules, which, in turn, may change the quantity of CBZ present on the electrode. Both components greatly contributed to improving the peak current, which reached its highest level when the pH was 7.0. Furthermore, when the pH increased to 10.0, there was a gradual reduction in the oxidation peak current. This could be attributed to the fact that CBZ becomes unstable and easily decomposed in an alkaline environment, and it is also influenced by deprotonation [33].

The relationship between the CBZ oxidation peak current and accumulation time was also investigated. As shown in Figure S2B, when the accumulation extended from 2 to 4 min, the peak current of CBZ oxidation showed a steady rise, and after 5 min, it finally reached its maximum value. The observed phenomenon can be explained by the fact that the Pt and rGO composites are electrically conductive and have high surface areas. This composite material showed a greater capacity for CBZ adsorption and facilitated enhanced electron transfer between CBZ and the modified GCE surface over prolonged periods. Therefore, there was a rise in oxidation peak current. However, the current signals exhibited a gradual decline as the time interval increased from 5 to 8 min (Figure S2C). As previously stated, the accumulation time had an impact on the current response, providing support for the hypothesis that CBZ molecules are partly adsorbed onto the Pt- and rGO-modified GCE surface. Furthermore, it is evident that the electrochemical response of CBZ is partially influenced by some CBZ accumulation phenomena on the surface of Pt-rGO/GCE.

### 3.5. Detection of CBZ via Differential Pulse Voltammetry (DPV)

**Figure 5:**
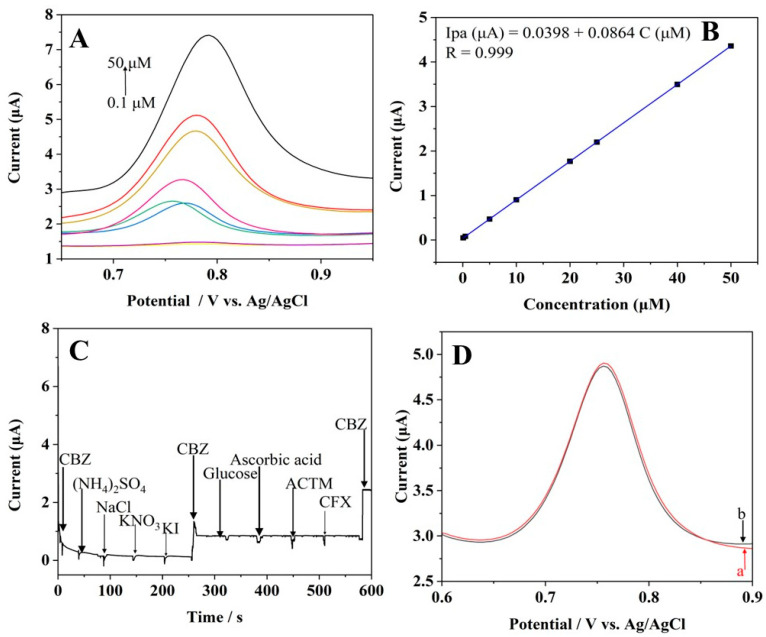
(**A**) DPV measurements of various concentrations of CBZ in pH of 7.0 PBS (0.1, 0.5, 5, 10, 20, 25, 40 and 50 µM) using Pt-rGO/GCE. (**B**) Calibration plot of CBZ. (**C**) Interference performance of 150 µM of (NH4)_2_SO_4,_ NaCl, KNO_3_, KI, glucose, ascorbic acid, acetaminophen (ACTM), and ciprofloxacin (CFX) in a solution containing 50 µM CBZ with pH of 7.0 PBS (+0.3 V). (**D**) DPV measurements of 50 µM CBZ in pH of 7.0 PBS at Pt-rGO/GCE: (a) initial test and (b) after 7 days of storage at room temperature.

An electrochemical approach of differential pulse voltammetry (DPV) was employed for CBZ detection under optimised experimental conditions, including a solution with a pH of 7.0 and an accumulation time of 5 min. Figure 5A depicts the DPV measurements of various concentrations of CBZ at Pt-rGO/GCE after 5 min of accumulation. When the concentration of CBZ varied from 0.1 to 50 µM, a linear increase in current responsiveness was seen. The equation for regression was presented as follows: Ipa (μA) = 0.0398 + 0.0864 C (μM) as evidenced by the correlation coefficient of 0.9999 (displayed in Figure 5B). Further, 3.46 nM was determined to be the minimum amount detectable by the sensor. 

Resistance ability in terms of potential interference with the developed Pt-rGO/GCE was examined when exposed to potential interferences, such as drugs, organic compounds, and inorganic salts. As shown in Figure 5C, 50 µM CBZ detection is not affected by the addition of 150 µM of inorganic salts such as (NH_4_)_2_SO_4_, NaCl, KNO_3_, KI, glucose, ascorbic acid, acetaminophen, and ciprofloxacin. The experimental findings indicate that Pt-rGO/GCE successfully mitigated the influence of potential interfering species and exhibited notable selectivity, resulting in sensing efficacy when detecting CBZ. To assess the stability of Pt-rGO/GCE, the developed sensor was preserved for 7 days at room temperature. The current intensity for CBZ only dropped by 2.06% after 7 days of storage, as illustrated in Figure 5D. To assess the reproducibility, the current response of five consecutive samples containing 50 µM CBZ was measured using DPV at the same time as Pt-rGO/GCE (Figure S2D). The relative standard deviation percentage (RSD %) was determined to be 0.692% indicating that the as-prepared sensor in this study exhibited a high level of reproducibility.

### 3.6. Analysis of CBZ in Real Samples 

The standard addition approach was applied to detect various levels of CBZ in tap water and skim milk samples using Pt-rGO/GCE, and the results are presented in Table 1. The CBZ concentration was determined using the calibration plot (Figure 5B). The findings indicate that the recoveries ranged between 98.40–99.36% and 97.94–99.21% for PBS: skim milk and PBS: tap water samples, respectively. Further, RSD was less than 3%. These findings confirm that Pt-rGO/GCE shows favourable reliability in terms of CBZ analysis in real samples.

## 4. Conclusions

The development of a Pt-rGO nanocomposite-modified electrode as an electrochemical sensor for detecting CBZ with high sensitivity was successfully achieved in this research study. The sensor was prepared through a simple and eco-friendly one-step electrodeposition process utilising the CV technique. The resulting electrode underwent various thorough analysis techniques, including SEM, EDX, CV, and EIS. Because of the synergistic effect from the combination of rGO sheets and distinctively structured Pt nanoparticles, the prepared Pt-rGO/GCE exhibited significantly improved electrochemical properties. As a result, the modified electrode effectively detected trace amounts of CBZ up to a limit of 3.46 nM with a linear range increasing from 0.1 µM to 50 µM using DPV. In addition, the presence of milk and tap water did not cause significant interference in the electrochemical detection process towards CBZ. With excellent stability, reproducibility, and selectivity noted in terms of the as-fabricated Pt-rGO/GCE. This method shows great promise in terms of the development of robust sensors for the detection of CBZ or similar pesticides in the future.

## Figures and Tables

**Table 1:** Detection of CBZ in tap water and skim milk (*n* = 3).

Mixture	Added (µM)	Found (µM)	Recovery (%)	RSD (%)
PBS:skim milk	25	24.60	98.40	2.80
	50	49.68	99.36	1.72
PBS:tap water	25	24.48	97.94	1.37
	50	49.60	99.21	1.10

## Data Availability

The data that support the findings of this study are available from the corresponding author upon reasonable request.

## Supplementary Materials

The following supporting information can be downloaded at: https://www.mdpi.com/article/10.3390/ma16247622/s1, Figure S1: EDX Spectra of (A) electrodeposited rGO, (B) electrodeposited Pt-rGO, and (C) electrodeposited Pt on GCE surface. (D) Pt particle size distribution histogram of the Pt and rGO modified GCE surface. (E) Pt particle size distribution histogram of the Pt modified GCE surface. Figure S2: (A) Closer view of CV graphs of bare GCE in (a) the absence of CBZ; and (b) bare GCE, and (c) rGO/GCE in the presence of 50 µM CBZ in pH 7.0 PBS at 0.1 V/s. (B) Effect of accumulation time of the 50 μM CBZ for varying periods of time 2, 3, 4, 5 min, and (C) 5, 6, 7, 8 min. (D) DPV measurements of 5 samples containing 50 µM CBZ with PBS (pH 7.0). Figure S3: The diagrammatic representation of electrochemical oxidation of CBZ..

## References

[1] Joseph X.B., Baby J.N., Wang S.-F., Sriram B., George M. (2021). Interfacial Superassembly of Mo2C@NiMn-LDH Frameworks for Electrochemical Monitoring of Carbendazim Fungicide. ACS Sustain. Chem. Eng..

[2] Kasaeinasab A., Mahabadi H.A., Shahtaheri S.J., Faridbod F., Ganjali M.R., Mesgari F. (2023). Carbendazim trace analysis in different samples by using nanostructured modified carbon paste electrode as voltametric sensor. PLoS ONE.

[3] Mahmoudi-Moghaddam H., Akbari Javar H., Garkani-Nejad Z. (2022). Fabrication of platinum-doped NiCo2O4 nanograss modified electrode for determination of carbendazim. Food Chem..

[4] Suresh I., Selvaraj S., Nesakumar N., Rayappan J.B.B., Kulandaiswamy A.J. (2021). Nanomaterials based non-enzymatic electrochemical and optical sensors for the detection of carbendazim: A review. Trends Environ. Anal. Chem..

[5] Nataraj N., Chen T.W., Akilarasan M., Chen S.M., Al-Ghamdi A.A., Elshikh M.S. (2022). Se substituted 2D-gC(3)N(4) modified disposable screen-printed carbon electrode substrate: A bifunctional nano-catalyst for electrochemical and absorption study of hazardous fungicide. Chemosphere.

[6] Huang S.M., Hu Y.F., Chen Y.L., Li G.K., Xia L. (2020). Magnetic solid-phase extraction coupled with high performance liquid chromatography for pesticide residues analysis in citrus sample. Chin. J. Anal. Chem..

[7] Tzatzarakis M., Kokkinakis M., Renieri E., Goumenou M., Kavvalakis M., Vakonaki E., Chatzinikolaou A., Stivaktakis P., Tsakiris I., Rizos A. (2020). Multiresidue analysis of insecticides and fungicides in apples from the Greek market. Applying an alternative approach for risk assessment. Food Chem. Toxicol..

[8] Chu Y., Tong Z., Dong X., Sun M., Gao T., Duan J., Wang M. (2020). Simultaneous determination of 98 pesticide residues in strawberries using UPLC-MS/MS and GC-MS/MS. Microchem. J..

[9] Mahdavi V., Ghorbani-Paji F., Ramezani M.K., Ghassempour A., Aboul-Enein H.Y. (2019). Dissipation of carbendazim and its metabolites in cucumber using liquid chromatography tandem mass spectrometry. Int. J. Environ. Anal. Chem..

[10] Umapathi R., Ghoreishian S.M., Sonwal S., Rani G.M., Huh Y.S. (2022). Portable electrochemical sensing methodologies for on-site detection of pesticide residues in fruits and vegetables. Coord. Chem. Rev..

[11] Pérez-Fernández B., Costa-García A., Muñiz A.d.l.E. (2020). Electrochemical (Bio)Sensors for Pesticides Detection Using Screen-Printed Electrodes. Biosensors.

[12] Santana P.C., Lima J., Santana T., Santos L.F., Matos C.R., da Costa L.P., Gimenez I.F., Sussuchi E.M. (2019). Semiconductor nanocrystals-reduced graphene composites for the electrochemical detection of carbendazim. J. Braz. Chem. Soc..

[13] Loguercio L.F., Thesing A., Demingos P., de Albuquerque C.D.L., Rodrigues R.S.B., Brolo A.G., Santos J.F.L. (2021). Efficient acetylcholinesterase immobilization for improved electrochemical performance in polypyrrole nanocomposite-based biosensors for carbaryl pesticide. Sens. Actuators B Chem..

[14] Liao X., Huang Z., Huang K., Qiu M., Fuliang C., Zhang Y., Wen Y., Chen J. (2019). Highly Sensitive Detection of Carbendazim and Its Electrochemical Oxidation Mechanism at a Nanohybrid Sensor. J. Electrochem. Soc..

[15] Gao X., Gao Y., Bian C., Ma H., Liu H. (2019). Electroactive nanoporous gold driven electrochemical sensor for the simultaneous detection of carbendazim and methyl parathion. Electrochim. Acta.

[16] Jo H.J., Shit A., Jhon H.S., Park S.Y. (2020). Highly sensitive non-enzymatic wireless glucose sensor based on Ni–Co oxide nanoneedle-anchored polymer dots. J. Ind. Eng. Chem..

[17] Patel M., Agrawal M., Srivastava A. (2022). Signal amplification strategies in electrochemical biosensors via antibody immobilization and nanomaterial-based transducers. Mater. Adv..

[18] Duc Le T., Ahemad M.J., Kim D.-S., Lee B.-H., Oh G.-J., Shin G.-S., Nagappagari L.R., Dao V., Van Tran T., Yu Y.-T. (2023). Synergistic effect of Pt-Ni dual single-atoms and alloy nanoparticles as a high-efficiency electrocatalyst to minimize Pt utilization at cathode in polymer electrolyte membrane fuel cells. J. Colloid Interface Sci..

[19] Venegas C.J., Bollo S., Sierra-Rosales P. (2023). Carbon-Based Electrochemical (Bio)sensors for the Detection of Carbendazim: A Review. Micromachines.

[20] Nia P.M., Abouzari-Lotf E., Woi P.M., Alias Y., Ting T.M., Ahmad A., Che Jusoh N.W. (2019). Electrodeposited reduced graphene oxide as a highly efficient and low-cost electrocatalyst for vanadium redox flow batteries. Electrochim. Acta.

[21] Pham T.S.H., Mahon P.J., Lai G., Fu L., Lin C.-T., Yu A. (2019). Cauliflower-like Platinum Particles Decorated Reduced Graphene Oxide for Sensitive Determination of Acetaminophen. Electroanalysis.

[22] Villalobos E., Marco J.F., Yáñez C. (2023). Reduced Graphene Oxide as a Platform for the Immobilization of Amino-Cyclodextrins. Micromachines.

[23] Noce R.D., Eugénio S., Siwek K.I., Silva T.M., Carmezim M.J., Sakita A.M.P., Lavall R.L., Montemor M.F. (2020). Direct electrodeposition of hydrogenated reduced graphene oxide from unsonicated solution and its electrochemical response. Diam. Relat. Mater..

[24] Peregrino P.P., Cavallari M.R., Fonseca F.J., Moreira S.G.C., Sales M.J.A., Paterno L.G. (2020). Starch-Mediated Immobilization, Photochemical Reduction, and Gas Sensitivity of Graphene Oxide Films. ACS Omega.

[25] Alinejadian N., Kazemi S.H., Nasirpouri F., Odnevall I. (2023). Electro-deposited nano-Ni/reduced graphene oxide composite film of corrugated surface for high voltammetric sensitivity. Mater. Chem. Phys..

[26] Sakthivel R., Lin L.Y., Duann Y.F., Chen H.H., Su C., Liu X., He J.H., Chung R.J. (2022). MOF-Derived Cu-BTC Nanowire-Embedded 2D Leaf-like Structured ZIF Composite-Based Aptamer Sensors for Real-Time In Vivo Insulin Monitoring. ACS Appl. Mater. Interfaces.

[27] Li G., Li W., Li S., Li X., Yao X., Xue W., Liang J., Chen J., Zhou Z. (2022). A label-free electrochemical aptasensor based on platinum@palladium nanoparticles decorated with hemin-reduced graphene oxide as a signal amplifier for glypican-3 determination. Biomater. Sci..

[28] Ateş A.K., Er E., Çelikkan H., Erk N. (2017). Reduced graphene oxide/platinum nanoparticles/nafion nanocomposite as a novel 2D electrochemical sensor for voltammetric determination of aliskiren. New J. Chem..

[29] Brett C.M.A. (2022). Electrochemical Impedance Spectroscopy in the Characterisation and Application of Modified Electrodes for Electrochemical Sensors and Biosensors. Molecules.

[30] Pham T.S.H., Hasegawa S., Mahon P., Guérin K., Dubois M., Yu A. (2022). Graphene Nanocomposites Based Electrochemical Sensing Platform for Simultaneous Detection of Multi-drugs. Electroanalysis.

[31] Feroze M.T., Doonyapisut D., Gudal C.C., Kim B., Chung C.-H. (2023). Impedimetric sensing platform for sensitive carbendazim detection using MOCVD-synthesized copper graphene. Mikrochim. Acta.

[32] de Macedo J.F., Alves A.A.C., Sant’Anna M.V.S., Cunha F.G.C., Oliveira G.d.A.R., Lião L.M., Sussuchi E.M. (2022). Electrochemical determination of carbendazim in grapes and their derivatives by an ionic liquid-modified carbon paste electrode. J. Appl. Electrochem..

[33] Yamuna A., Chen T.W., Chen S.M., Jiang T.Y. (2021). Facile synthesis of single-crystalline Fe-doped copper vanadate nanoparticles for the voltammetric monitoring of lethal hazardous fungicide carbendazim. Mikrochim. Acta.

