# Supplementary material for: Facile Synthesis of Platinum Nanoparticle-Embedded Reduced Graphene Oxide for the Detection of Carbendazim
Source: Materials (Basel). 2023 Dec 13;16(24):7622. doi: 10.3390/ma16247622 (PMC10744356; doi:10.3390/ma16247622)
Supplement: Supplementary file 1 [file materials-16-07622-s001.zip › materials-2755199-supplementary.pdf]

Supporting Information

Facile Synthesis of Platinum Nanoparticle-Embedded Reduced Graphene Oxide for the Detection of Carbendazim

Suthira Pushparajah, Shinichi Hasegawa †, Tien Song Hiep Pham, Mahnaz Shafiei and Aimin Yu *

School of Science, Computing, and Engineering Technology, Swinburne University of Technology, Hawthorn, VIC 3122, Australia
* Correspondence: aiminyu@swin.edu.au
† Current address: Japan Patent Office, Chiyoda-ku, Tokyo 100-8915, Japan.

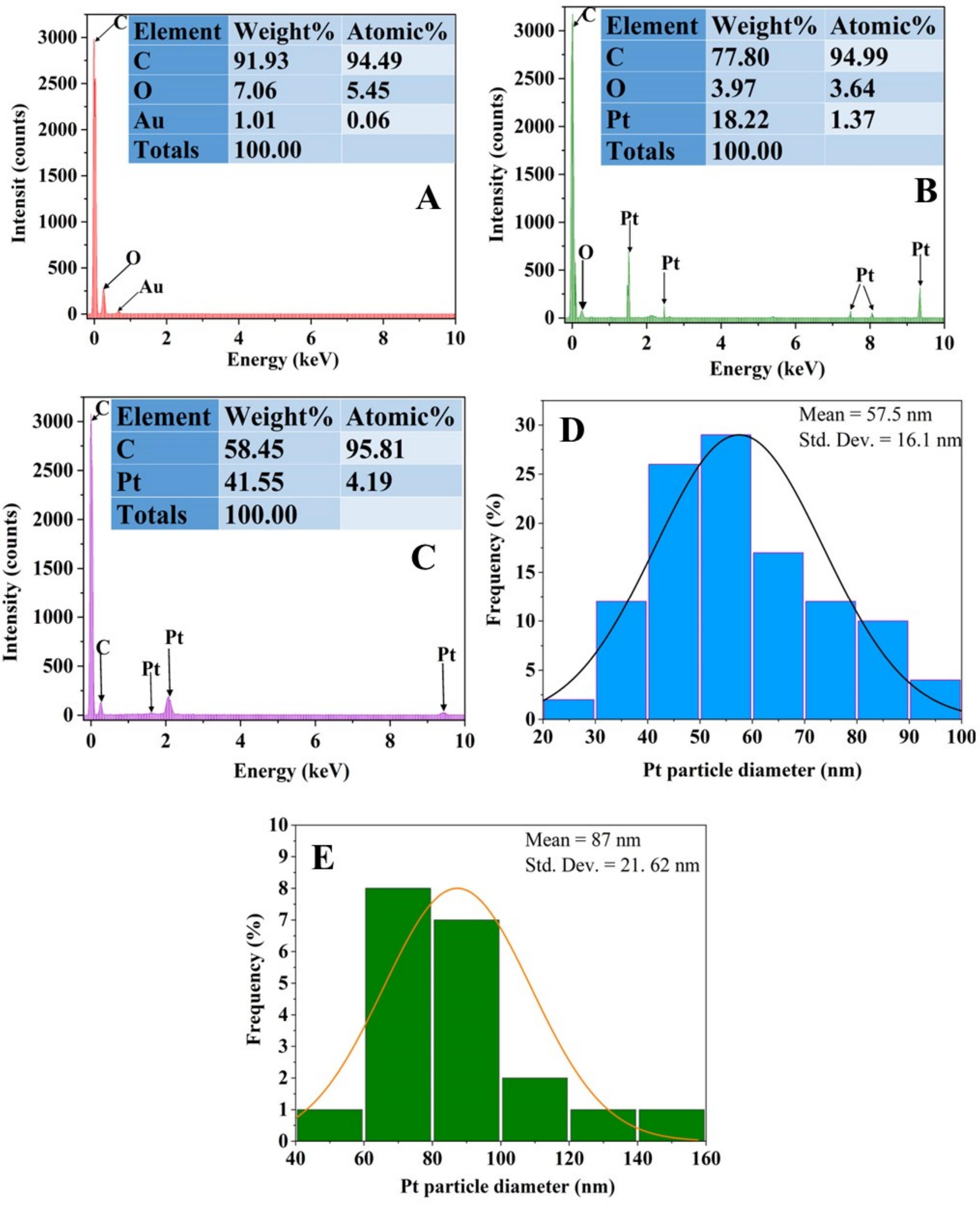

Figure S1. EDX Spectra of (A) electrodeposited rGO, (B) electrodeposited Pt-rGO, and (C) electrodeposited Pt on GCE surface. (D) Pt particle size distribution histogram of the Pt and rGO modified GCE surface. (E) Pt particle size distribution histogram of the Pt modified GCE surface.

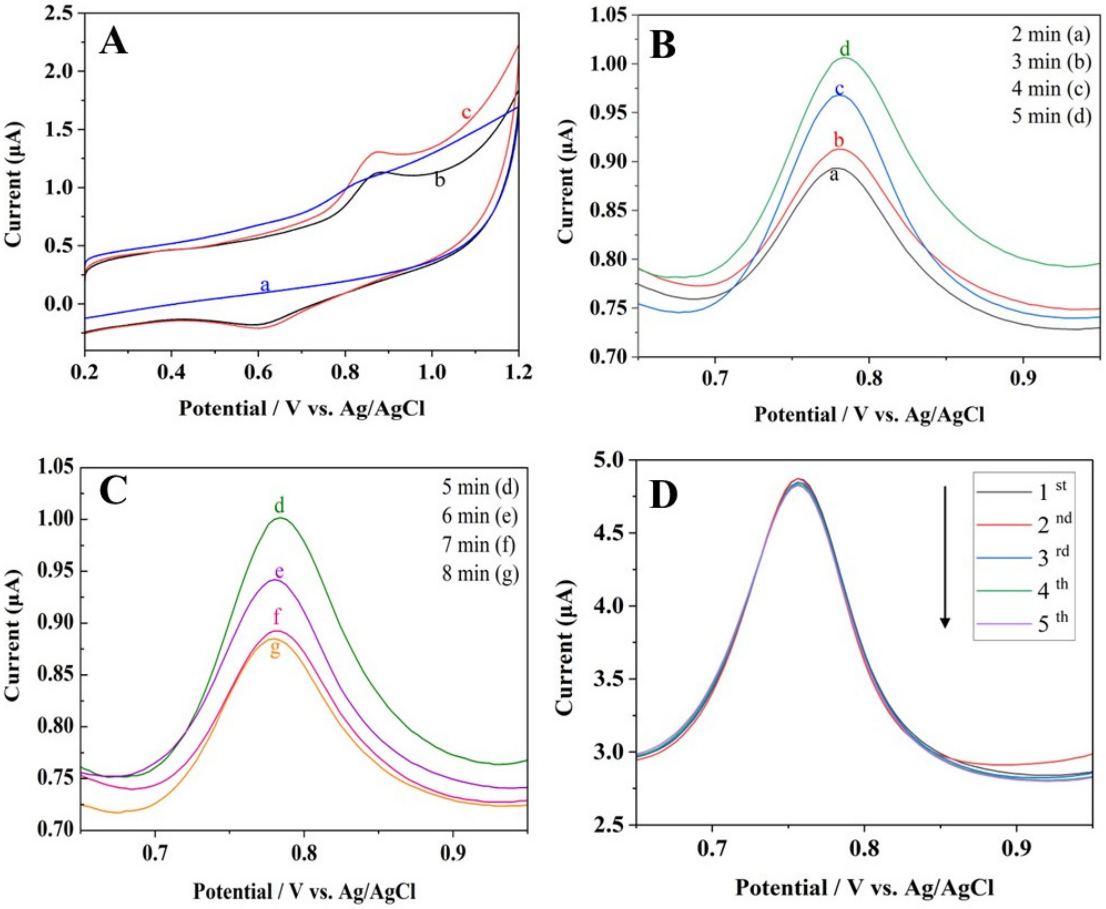

Figure S2. (A) Closer view of CV graphs of bare GCE in (a) the absence of CBZ; and (b) bare GCE, and (c) rGO/GCE in the presence of 50 µM CBZ in pH 7.0 PBS at 0.1 V/s. (B) Effect of accumulation time of the 50 µM CBZ for varying periods of time 2, 3, 4, 5 min, and (C) 5, 6, 7, 8 min. (D) DPV measurements of 5 samples containing 50 µM CBZ with PBS (pH 7.0).

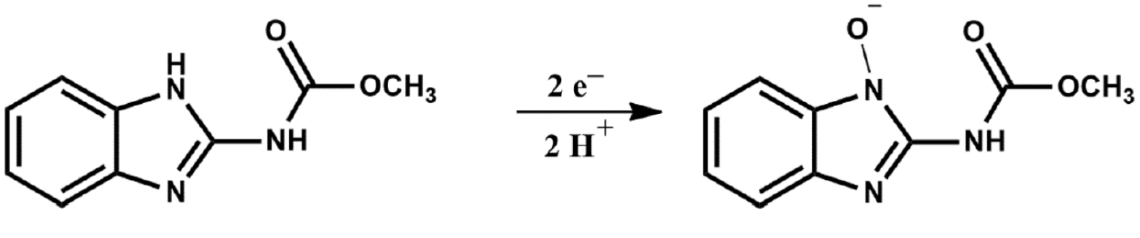

Figure S3. The diagrammatic representation of electrochemical oxidation of CBZ [1].

References

1. de Macedo, J.F.; Alves, A.A.C.; Sant'Anna, M.V.S.; Cunha, F.G.C.; Oliveira, G.d.A.R.; Lião, L.M.; Sussuchi, E.M. Electrochemical determination of carbendazim in grapes and their derivatives by an ionic liquid-modified carbon paste electrode. *J. Appl. Electrochem.* **2022**, *52*, 729-742.
